# Effect of VEGF receptor inhibitor PTK787/ZK222548 combined with ionizing radiation on endothelial cells and tumour growth

**DOI:** 10.1054/bjoc.2001.2166

**Published:** 2001-12

**Authors:** C Hess, V Vuong, I Hegyi, O Riesterer, J Wood, D Fabbro, C Glanzmann, S Bodis, M Pruschy

**Affiliations:** Department of Radiation Oncology, Department of Pathology, University Hospital Zurich, Zurich, CH-8091, Switzerland; Department of Oncology Research, Novartis Pharma Inc., Basel, CH-4002, Switzerland

**Keywords:** angiogenesis, radiosensitization, VEGF-dependent proliferation, tumour growth control

## Abstract

The vascular endothelial growth factor (VEGF) receptor is a major target for anti-angiogenesis-based cancer treatment. Here we report the treatment effect of ionizing radiation in combination with the novel orally bioavailable VEGF receptor tyrosine kinase inhibitor PTK787/ZK222584 on endothelial cell proliferation in vitro and with tumour xenografts in vivo. Combined treatment of human umbilical vein endothelial cells with increasing doses of PTK787/ZK222584 and ionizing radiation abrogated VEGF-dependent proliferation in a dose-dependent way, but inhibition of endothelial cell proliferation was not due to apoptosis induction. In vivo, a combined treatment regimen of PTK787/ZK222584 (4 × 100 mg/kg) during 4 consecutive days in combination with ionizing radiation (4 × 3 Gy) exerted a substantial tumour growth delay for radiation-resistant p53-disfunctional tumour xenografts derived from SW480 colon adenocarcinoma cells while each treatment modality alone had only a minimal effect on tumour size and neovascularization. SW480 tumours from animals that received a combined treatment regimen, displayed not only an extended tumour growth delay but also a significant decrease in the number of microvessels in the tumour xenograft. These results support the model of a cooperative antitumoural effect of angiogenesis inhibitor and irradiation and show that the orally bioavailable VEGF receptor tyrosine kinase inhibitor PTK787/ZK222584 is suitable for combination therapy with irradiation. © 2001 Cancer Research Campaign http://www.bjcancer.com


British Journal of Cancer (2001) 85(12), 2010–2016
© 2001 Cancer Research Campaign
doi: 10.1054/ bjoc.2001.2166, available online at http://www.idealibrary.com on IDEAL
http://www.bjcancer.com

### Effect of VEGF receptor inhibitor PTK787/ZK222548 combined with ionizing radiation on endothelial cells and tumour growth

**C Hess[1], V Vuong[1], I Hegyi[2], O Riesterer[1], J Wood[3], D Fabbro[3], C Glanzmann[1], S Bodis[1] and M Pruschy[1]**

[1]Department of Radiation Oncology; [2]Department of Pathology, University Hospital Zurich, CH-8091 Zurich, Switzerland; [3]Novartis Pharma Inc., Department of Oncology Research, CH-4002 Basel, Switzerland

**Summary** The vascular endothelial growth factor (VEGF) receptor is a major target for anti-angiogenesis-based cancer treatment. Here we report the treatment effect of ionizing radiation in combination with the novel orally bioavailable VEGF receptor tyrosine kinase inhibitor PTK787/ZK222584 on endothelial cell proliferation in vitro and with tumour xenografts in vivo. Combined treatment of human umbilical vein endothelial cells with increasing doses of PTK787/ZK222584 and ionizing radiation abrogated VEGF-dependent proliferation in a dose-dependent way, but inhibition of endothelial cell proliferation was not due to apoptosis induction. In vivo, a combined treatment regimen of PTK787/ZK222584 ($4 \times 100$ mg/kg) during 4 consecutive days in combination with ionizing radiation ($4 \times 3$ Gy) exerted a substantial tumour growth delay for radiation-resistant p53-disfunctional tumour xenografts derived from SW480 colon adenocarcinoma cells while each treatment modality alone had only a minimal effect on tumour size and neovascularization. SW480 tumours from animals that received a combined treatment regimen, displayed not only an extended tumour growth delay but also a significant decrease in the number of microvessels in the tumour xenograft. These results support the model of a cooperative antitumoural effect of angiogenesis inhibitor and irradiation and show that the orally bioavailable VEGF receptor tyrosine kinase inhibitor PTK787/ZK222584 is suitable for combination therapy with irradiation. © 2001 Cancer Research Campaign http://www.bjcancer.com

**Keywords**: angiogenesis; radiosensitization; VEGF-dependent proliferation; tumour growth control

The formation of new vessels from an existing vascular network in the tumour area is essential for tumour progression and the formation of metastasis (Folkman, 1995). Therefore inhibition of angiogenesis is a promising cancer treatment that is directed against the delivery of nutrients, growth factors and oxygen supply to the tumour.

Of the numerous pro- and anti-angiogenic growth factors and cytokines, vascular endothelial growth factor (VEGF) is an important pro-angiogenic factor in pathological situations that involve neovascularization and enhanced vascular permeability (Hanahan and Folkman, 1996). Evidence for the importance of VEGF-induced angiogenesis in tumour growth includes the observation that neutralizing antibodies against VEGF or dominant negative soluble receptors inhibit the growth of primary and metastatic experimental tumours (Kim et al, 1993; Asano et al, 1995; Borgestrom et al, 1996; Goldman et al, 1998; Kanai et al, 1998).

The VEGF receptors Flt-1 (Fms-like tyrosine kinase, VEGF-R1) and KDR (kinase insert domain-containing receptor, VEGF-R2) are almost exclusively located on endothelial cells (Shibuya et al, 1990; Terman et al, 1992). Expression of these receptors is low in normal tissues but they are upregulated when neovascularization occurs (Brown et al, 1993a,b). Both receptors have seven immunoglobulin-like domains in their extracellular region, a single transmembrane-spanning domain, and an intracellular split tyrosine kinase domain, and belong to the same family of receptors as EGFR, PDGFR, c-Kit, c-Fms, Flt-3 and Flt-4. Flt-1 binds VEGF-A and -B (Olofsson et al, 1996, 1998) and the related placental growth factor (Park et al, 1994; Clauss et al, 1996), whereas KDR binds VEGF-A, -C and -D (Joukov et al, 1997; Yamada et al, 1997). In contrast to Flt-1, KDR is strongly autophosphorylated upon VEGF stimulation and mediates the mitogenic response (Takahashi and Shibuya, 1997; Kroll and Waltenberger, 1997). Therefore, targeting the tyrosine kinase of the KDR VEGF receptor by small pharmacological inhibitors represents a promising anti-angiogenic strategy for treatment of solid tumour malignancies (Fong et al, 1999; Wood et al, 2000).

Whereas anti-angiogenic strategies are aimed in the first line against the endothelial vascular system, treatment of tumours with ionizing radiation (IR) directly targets tumour tissue. Interestingly, recent results with combined treatment using angiostatin, a proteolytic fragment of plasminogen (Mauceri et al, 1998; Gorski et al, 1998) and neutralizing anti-human $VEGF_{165}$ antibodies (Gorski et al, 1999), as anti-angiogenic factors, in combination with IR demonstrated a potentiated cytotoxic effect against endothelial cells and an increased anti-tumoural effect of the combination therapy in vivo. Phase I studies with angiostatin in combination with IR are currently in progress. Likewise combined treatment with anti-VEGFR2 monoclonal antibodies or the anti-angiogenic compound TNP-470 significantly enhanced the growth-retarding effect of irradiation (Teicher et al, 1996; Lund et al, 2000; Kozin et al, 2001).

In this report we tested the effect of combined treatment of IR in combination with the novel, orally bioavailable VEGF receptor

*Received 16 July 2001*
*Revised 2 October 2001*
*Accepted 4 October 2001*

*Correspondence to:* M Pruschy tyrosine kinase inhibitor PTK787/ZK222584 on endothelial cell proliferation in vitro and demonstrate potent growth inhibition of human SW480 adenocarcinoma tumour xenografts in an in vivo mouse model by combination therapy. PTK787/ZK222584 is an aminophthalazine derivative and is currently under clinical investigation (Wood et al, 2000; Drevs et al, 2000).

### MATERIALS AND METHODS

### Materials

PTK787/ZK222584 (1-[4-Chloroanilino]-4-[4-pyridylmethyl]-phthalazine succinate) was obtained from Novartis Pharma, Switzerland. For in vitro assays, a stock solution of 10 mM of PTK787/ZK222584 was prepared in DMSO and further diluted in serum-containing media. Human $VEGF_{165}$ was obtained from R&D Systems. The final concentration of VEGF in all experiments was 10 ng/ml.

### Cell cultures and irradiation

Human umbilical vein endothelial cells (HUVEC, PromoCell) were used between the fourth and the eighth passage number and cultured for optimal cell growth conditions at 37˚C and 5% $CO_2$ atmosphere in complete endothelial cell growth medium (EGM, PromoCell GmbH) containing 0.4% ECGS/H, 0.1 ng/ml EGF, 1 µg/ml hydrocortisone, 1 ng/ml bFGF, 5% fetal calf serum, 50 ng/ml amphotericin B and penicillin/streptomycin. For growth factor starvation, cells were incubated in basal endothelial cell media (PromoCell GmbH) supplemented with 1.5% fetal calf serum (EBM) to minimize cell proliferation but still guarantee cell survival. VEGF-triggered cell proliferation was induced by addition of VEGF (10 ng/ml or otherwise indicated) to EBM, i.e. in the presence of 1.5% fetal calf serum. Before use, all tissue culture dishes and 96-well plates were coated with 1.5% gelatin and washed with PBS buffer, except where otherwise noted. SW480 colon adenocarcinoma cells were cultured in RPMI-1640 media (Gibco BRL) supplemented with 10% fetal calf serum and penicillin/streptomycin. Irradiation of cell cultures was carried out at RT in tissue culture dishes (100 × 100 mm) or in 96-well plates using a Pantak Therapax 300 kV X-ray unit at 0.7 Gy/min. Dosimetry was controlled with a Vigilant dosimeter.

### Cell proliferation and BrdU-incorporation assay

Human umbilical vein endothelial cells (HUVECs) ($1 \times 10^3$ cells/well) were seeded in 96-well plates in EBM and allowed to attach overnight. Pre-incubation with PTK787/ZK222584 was performed for 2 h prior to h-$VEGF_{165}$ (10 ng/ml) stimulation and followed by irradiation 2 h later. Proliferation was assessed at the indicated timepoints with the colorimetric AlamarBlue assay that is based on detection of metabolite activity according to the protocol of the manufacturer (Biosource International). Absorption was measured at 570 nm and 600 nm using a Dynatech MR5000 spectrophotometer. Proliferation experiments with SW480 cells were performed with $2 \times 10^3$ cells/well in RPMI-1640 media supplemented with 1.5% FCS. BrdU (5-Bromo-2′-deoxy-uridine)-incorporation was performed with a BrdU-ELISA-kit (5-Bromo-2′-deoxy-uridine Labeling and Detection Kit III; Boeringer Mannheim). HUVECs ($1 \times 10^3$ cell/well) were plated in EBM and treated as described for the proliferation assay (see earlier). Cells were incubated for 24 h followed by addition of BrdU labelling solution and a 2nd incubation was carried out over 24 h prior to fixation, blocking and addition of peroxidase-labelled anti-BrdU antibody. Bound antibody was detected using a colorimetric substrate and spectrophotometrically quantified at 405 nm and 490 nm using a Dynatech MR5000 spectrophotometer. Experiments were performed in triplicate and repeated at least three times.

### Antibodies and Western blots

HUVECs were detached from the petri dish using trypsin/EDTA, washed in PBS, and cell pellets were immediately frozen at – 70˚C. The protein concentration of whole-cell lysates was determined with the BioRad DC-protein assay. Cellular proteins (50–100 µg) were resolved by SDS-polyacrylamide gel electrophoresis and blotted onto PVDF membranes. Rabbit polyclonal anti-cleaved caspase-3 antibody was obtained from New England Biolabs. Antibody detection was achieved by ECL-enhanced chemiluminescence (Amersham) using a horseradish peroxidase-conjugated 2nd antibody, according to the manufacturer's protocol.

### Tumour xenografts in nude mice and administration of chemotherapy and irradiation

Human colon carcinoma cells (SW480) were injected subcutaneously ($4 \times 10^6$ cells) into the flank of 4–8-week-old athymic nude mice. Tumour volumes were determined from caliper measurements of tumour length (L) and width (l) according to the formula ($L \times l^2$)/2. Tumours were allowed to expand to a volume of at least 0.175 $cm^3$ (+/– 10%) before treatment. Using a customized shielding device, mice were given a strictly locoregional radiotherapy of 4 × 3 Gy using a Pantak Therapax 300 kV X-ray unit at 0.7 Gy/min. PTK787/ZK222584 (dissolved in 5% DMSO, 1% Tween-80 and 94% $H_2O$) was applied p.o. 0.5 h prior to irradiation at the indicated dosage. Statistical analysis was performed with the Mann–Whitney U-test. The absolute tumour growth delay (AGD) was defined as the time for tumour volume in the treated groups to triplicate the initial treatment size minus the time in the untreated control group to reach the same size (Sun et al, 1998). All animal experiments were performed according to the ethical standards required by the UKCCCR Guidelines (UKCCCR, 1998).

### Histological analysis

The resected tumours were fixed in 4% buffered formalin and embedded in paraffin for morphological analysis. Tumour sample sections of 4–8 µm embedded in paraffin were dewaxed with xylene, rehydrated with hematoxylin and eosin (H&E) and the endothelial cell-specific anti-CD31 antibody M083 (PECAM-1, Dako) to evaluate tissue structure, quantity, morphology and size of blood vessels.

### RESULTS

### Antiproliferative effect of PTK787/ZK222584 and IR in endothelial cells

The antiproliferative effect of the novel orally active VEGF receptor tyrosine kinase inhibitor PTK787/ZK222584 alone and in

© 2001 Cancer Research Campaign combination with IR was tested with primary human umbilical vein endothelial cells (HUVECs). Assessment of BrdU incorporation was used to test a short-term functional effect on cell proliferation after treatment with increasing concentrations of PTK787/ZK222584 (10–500 nM) alone and in combination with IR (5, 10 Gy) using basal medium (EBM) and VEGF- supplemented medium. HUVECs growing in EBM have a minimal proliferation rate but the presence of 1.5% fetal calf serum still guarantees cell survival (see Materials and methods). Cells were incubated following treatment for 48 h and BrdU incorporation was determined during the final 24 h of the incubation period (Figure 1, A and B).

The basal level of HUVEC proliferation in minimal EBM conditions supplemented with 1.5% FCS but in absence of VEGF corresponded to 20% of the BrdU incorporation observed in VEGF-stimulated cells. Increasing concentrations of PTK787/ZK222584 did not affect the basal level of BrdU incorporation in the absence of VEGF, but induced a dose-dependent decrease of proliferation in HUVECs when stimulated with VEGF. BrdU incorporation was reduced to basal levels when cells were treated with PTK787/ZK222584 concentrations above 100 nM. These results demonstrate the specific anti-VEGF-receptor-directed growth inhibitory effect of PTK787/ZK222584 (Figure 1B). Irradiation of HUVECs with 5 Gy or 10 Gy in the absence of VEGF even abrogated the basal proliferative activity, indicative of a complete IR-induced arrest of cell proliferation. When cells were grown in the presence of VEGF, irradiation with 5 Gy and 10 Gy decreased the amount of BrdU incorporation to 42% and 10%,

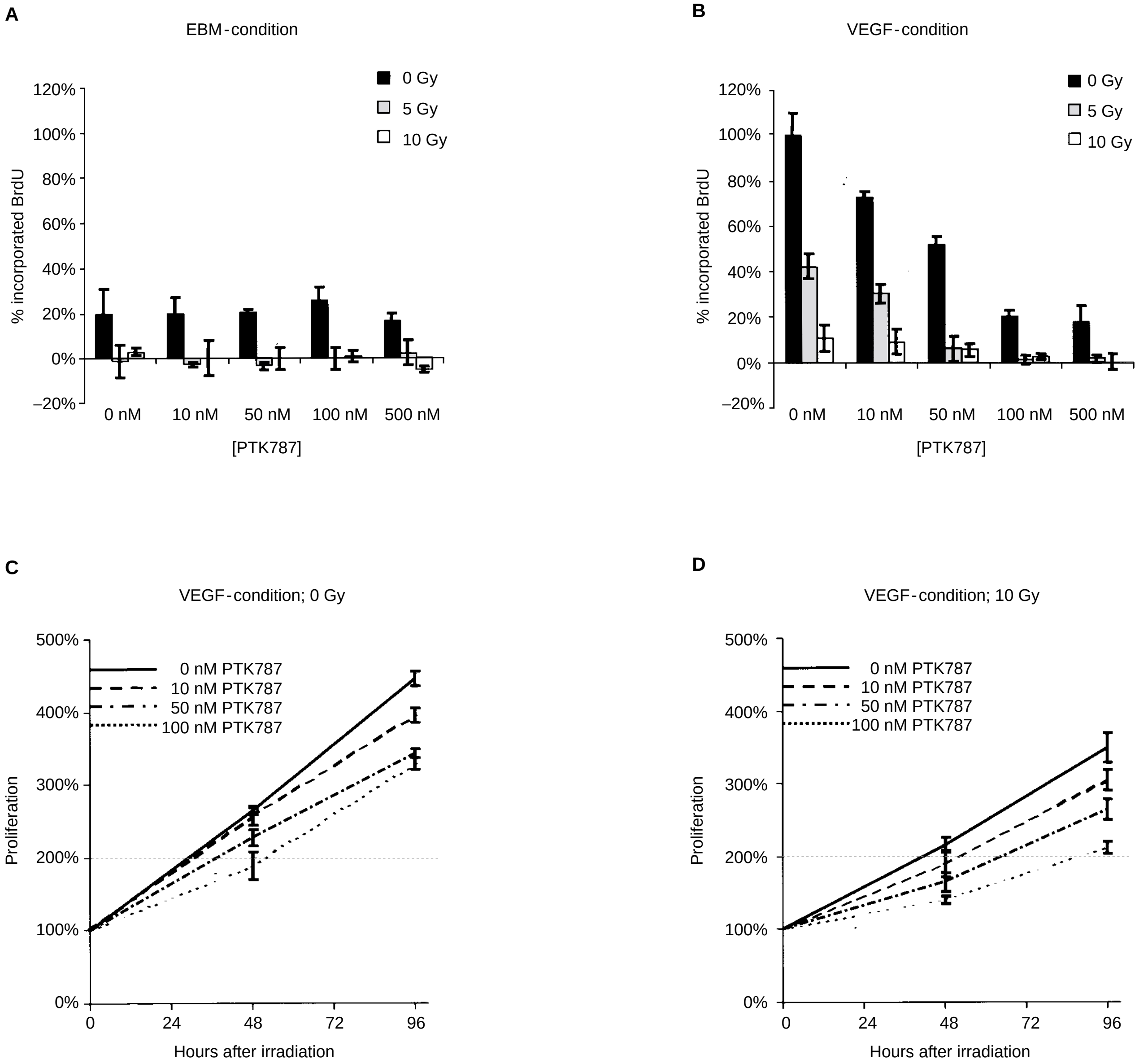

**Figure 1** Treatment with PTK787/ZK222584 alone and in combination with IR decreases the VEGF-dependent proliferative activity of HUVECs. HUVECs were treated with increasing doses of PTK787/ZK222584 and IR alone and in combination, in the absence (**A**) or presence of VEGF (**B**). BrdU-incorporation was determined 24 h after treatment during an additional 24-hour incubation period. The negative values are due to unspecific but enhanced BrdU/anti-BrdU antibody detection in control samples without cells. The prolonged antiproliferative effect on HUVECs in the presence of VEGF after treatment with increasing doses of PTK787/ZK222584 alone (**C**) and in combination with IR (**D**) was determined 48 h and 96 h with the MTT-like alamarblue assay. The proliferative activity of the cells prior to treatment was determined and set as 100%. Data show the mean of one representative experiment ± SD

© 2001 Cancer Research Campaign respectively. Combined treatment with PTK787/ZK222584 and irradiation showed an additive antiproliferative effect on VEGF-stimulated BrdU incorporation at low PTK787/ZK222584 concentrations (10, 50 nM).

The proliferative activity of HUVECs upon treatment with increasing concentrations of PTK787/ZK222584 and IR alone and in combination, was also determined at later timepoints (48 h and 96 h) using the MTT-like colorimetric alamarBlue assay that is based on detection of metabolic activity. Similar to the short-term proliferation assay, the dose-dependent effect of PTK787/ZK222584 on HUVEC proliferation was still observed at later timepoints (Figure 1C). Likewise irradiation (10 Gy) alone resulted in a decreased metabolic activity that corresponded with 66% of untreated cells at the 48 h and 96 h timepoints. Combined treatment with PTK787/ZK222584 and IR resulted in an additive reduction of proliferative activity determined at these timepoints (Figure 1D).

### No apoptosis induction by combined treatment

HUVECs undergo apoptosis after loss of adhesion or serum deprivation. The reduced proliferative activity observed in the HUVEC population upon treatment with PTK787/ZK222584 alone or in combination with IR might be due to the induction of apoptosis. However no morphological signs of apoptosis were observed during the course of treatment (data not shown). In order to detect any biochemical parameters of apoptosis induction, processing of the major effector caspase-3 was detected in cellular extracts by Western blotting with anti-cleaved caspase-3 antibody. This antibody only recognizes the active form of caspase-3. Treatment of cells that were incubated in the presence of VEGF-containing EBM media supplemented with 1.5% fetal calf serum, with PTK787/ZK222584 (100 nM), IR (10 Gy) or in combination did not induce any processing of caspase-3 to its active form, while cultivation of cells in EBM media in the absence of 1.5% serum and VEGF resulted in caspase-3 processing (Figure 2A).

The cellular response of HUVECs depends highly on the experimental conditions, e.g. absence or minimal serum present during treatment. Likewise no apoptosis induction was observed in the presence of minimal serum (1.5%) and VEGF upon combined treatment with IR and PTK787/ZK222584, at concentrations that induced apoptosis by treatment with PTK787/ZK222584 alone but in absence of serum (Wood et al, 2000).

Control experiments were performed with HUVECs cultivated in the presence of increased serum levels (10%). PTK787/ZK222584 did not decrease the highly proliferative activity observed under these conditions, which is most probably due to the high abundance of growth stimulatory factors in serum (data not shown).

### Treatment of SW480 tumour xenografts with IR and PTK787/ZK222584

The low molecular weight synthetic inhibitor PTK787/ZK222584 is active after oral administration and is currently undergoing clinical trial in cancer patients. Based on the additive in vitro effects, combined treatment with PTK787/ZK222584 and fractionated irradiation was tested in vivo against tumour xenografts derived from the p53-mutated, human colon adenocarcinoma cell line SW480. To exclude a direct effect of PTK787/ZK222584 on the SW480 tumour cells, control experiments were performed to test the anti-proliferative effect of PTK787/ZK222584 on this cell line. Treatment with increasing concentration of PTK787/ZK222584 had no effect on the proliferation of these cells consistent with the absence of KDR receptor expression in this cell line. Combined treatment with PTK787/ZK222584 and irradiation in vitro did not result in any additional inhibitory effect of PTK787/ZK222584 over the response induced by irradiation alone (Figure 2B).

Tumour xenografts were obtained upon subcutaneous injection of SW480 cells ($4 \times 10^6$) into the flank of nude mice and treatment was started when tumours reached a minimal size of 175 $mm^3$ ± 10%

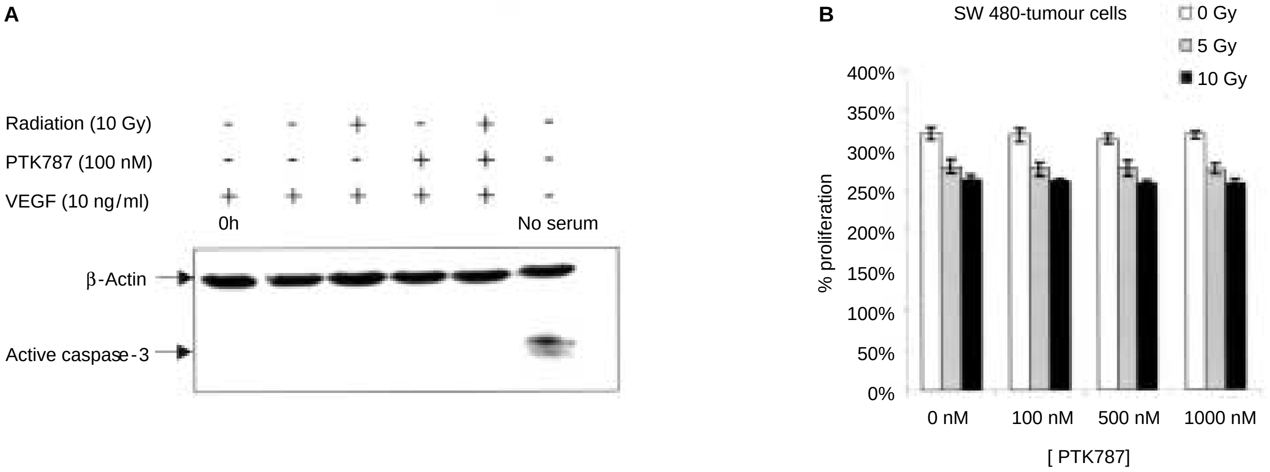

**Figure 2** PTK787/ZK222584 does not induce apoptosis in HUVECs and has no antiproliferative effect on SW480 cells. (**A**) HUVECs in EBM (1.5% FCS) and EBM supplemented with VEGF (10 ng/ml) were treated with PTK787/ZK222584 (100 nM), IR (10 Gy) and in combination. Cells were harvested 48 h after treatment and analysed for caspase-3 processing. The first lane is a control sample taken at the 0 h timepoint of cultivation in the specified media. Equal protein loading was verified with an anti-β-actin monoclonal antibody. Cell lysate from HUVECs cultivated in the absence of serum and VEGF was co-loaded as an apoptotic control sample. (**B**) Proliferation of SW480 cells, in RPMI-1640, 1.5% FCS and VEGF (10 ng/ml), was assessed 96 h after treatment with increasing doses of PTK787/ZK222584 alone and in combination with IR. The proliferative activity of the cells prior to treatment was determined and set as 100%

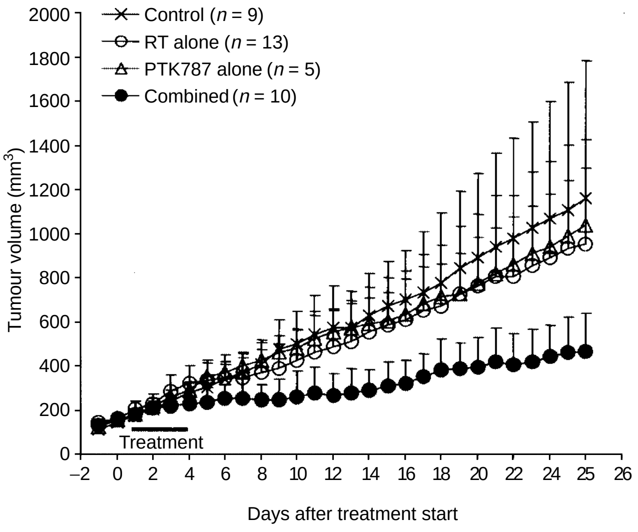

**Figure 3** The effect of PTK787/ZK222584 and RT alone or combined on the growth of human SW480 colon adenocarcinoma xenografts in nude mice. Mice were treated with PTK787/ZK222584 alone (4 × 100 mg/kg), IR alone (4 ×3 Gy), and in combination applied on 4 consecutive days, in comparison with an untreated control group. Each curve represents the mean tumour volume per group (*n* = quantity of animals per group) ± SD

(days 12–17 after cell injection). Figure 3 summarizes the effect of treatment against SW480-derived tumours with PTK787/ZK222584 alone (4 × 100 mg/kg), IR alone (4 × 3 Gy), and in combination (4 × 100 mg/kg combined with 4 × 3 Gy) applied on 4 consecutive days, in comparison with an untreated control group. Each curve represents the mean tumour volume per group (*n* = quantity of animals per group). A daily dose of 3 Gy was chosen as a clinically relevant fraction size for the treatment of human malignancies. For practical reasons, only four fractions were chosen as a treatment regimen, but the response to such a regimen is useful for evaluation of radiosensitization and treatment. Treatment with PTK787/ZK222584 or IR alone resulted in minimal growth delay, whereas combined treatment exerted a strong tumour growth control during treatment and the follow-up period. In this human colon tumour xenograft, a significant AGD to triplicate the tumour volume was observed upon combined treatment in comparison to the AGD upon treatment with IR or PTK787/ZK222584 alone (21 days vs 2 or 1 days, respectively) resulting in prolonged tumour growth control ($P = 0.003$, RT vs combined treatment). Examination of the tumours during and after the different treatments revealed that tumour tissue appeared to be much less vascularized (white-shining) after combined treatment in comparison to untreated tumours or after either treatment modality alone (data not shown).

### Histological analysis of microvessel density

Histopathological examination of control SW480 tumours revealed a dense network of distended microvessels localized in the central part of the tumour (Figure 4). Moreover a large number of vital microvessels were located at the border and focally in the necrotic and apoptotic tumour areas. In contrast, SW480 tumours with the combined therapy displayed a dramatically decreased number of microvessels, often with only a very thin diameter located almost exclusively in the vital parts of the tumours. SW480 tumours treated either with PTK787/ZK222584 or IR alone showed comparable microvessel density to the control group. Tumours of all treatment groups revealed extensive necrosis and apoptosis, usually only with a thin wall of vital tumour at the periphery. Owing to a dramatically decreased tumour volume after combined treatment, the total area of necrotic and apoptotic zones appears relatively enlarged in comparison to control tumours.

### DISCUSSION

Radiotherapy is an important treatment modality for many solid human cancers but often unsuccessful because of an intrinsic or acquired radiation resistance of the tumour. Solid tumours require angiogenesis for growth, and inhibition of angiogenesis is one promising strategy for cancer therapy. Therefore a combined treatment approach using pharmacological inhibitors of angiogenesis and ionizing radiation has a sound rationale for both targets within a solid tumour and the potential lack of supra-additive toxicity.

Here we have demonstrated the combined treatment modality of irradiation with PTK787/ZK222584, a specific low molecular inhibitor of the VEGF receptor tyrosine kinase that is currently under clinical investigation for its anti-angiogenic activity and is active upon oral application. In vitro, combined treatment with PTK787/ZK222584 and irradiation against human umbilical vein endothelial cells resulted in an additive antiproliferative effect. In comparison to other reports, we could not detect a supra-additive cytotoxic in vitro effect on endothelial cell proliferation as observed upon treatment with irradiation in combination with an angiogenic inhibitor in clonogenic assays (Mauceri et al, 1998; Gorski et al, 1999). Furthermore no apoptosis induction was observed in our experimental set-up. Our results indicate that the antiproliferative effect induced with PTK787/ZK222584 or IR as single agents or the combination of both might be due to a transient cell cycle arrest as detected by the abrogated BrdU incorporation at the early timepoints and a subsequent lower level of proliferative activity in the cell population at the later timepoints.

In vivo, combined treatment with four daily fractions of low-dose ionizing irradiation induced a strong cooperative growth control effect at doses of IR and PTK787/ZK222584 that had almost no effect when used as a single agent alone. These results are consistent with earlier observations that VEGF-dependent growth is a key factor for in vivo tumour neovascularization and a potential target for combined treatment modalities (Shibuya, 1995; Takahashi et al, 1995; Mauceri et al, 1998; Gorski et al, 1999; Kozin et al, 2001). With a treatment regimen of four fractions of IR applied on four consecutive days in vivo, we found both a significant suppra-additive, antitumoral effect and a lack of additive toxicity, indicating a potentially broad therapeutic window. We did not observe any immediate or long-term unspecific toxicities during treatment and the follow-up period. The results obtained with low doses of both irradiation and PTK787/ZK222584 are encouraging with regard to the translation of the findings into potential phase I studies for cancer patients. Thus, it would be interesting to test an extended combined treatment regimen against these tumour xenografts, in particular since the daily dose and number of fractions of IR and dose of PTK787/ZK222584 is well below the maximal daily tolerated dose of IR or PTK787/ZK222584 that could be given over an extended period of time (Wood et al, 2000).

Recently, inhibition of the VEGF receptor by the Flk-1 kinase inhibitor SU5416 was demonstrated to revert radiation resistance of refractory tumour blood vessels and enhanced the cytotoxic

© 2001 Cancer Research Campaign

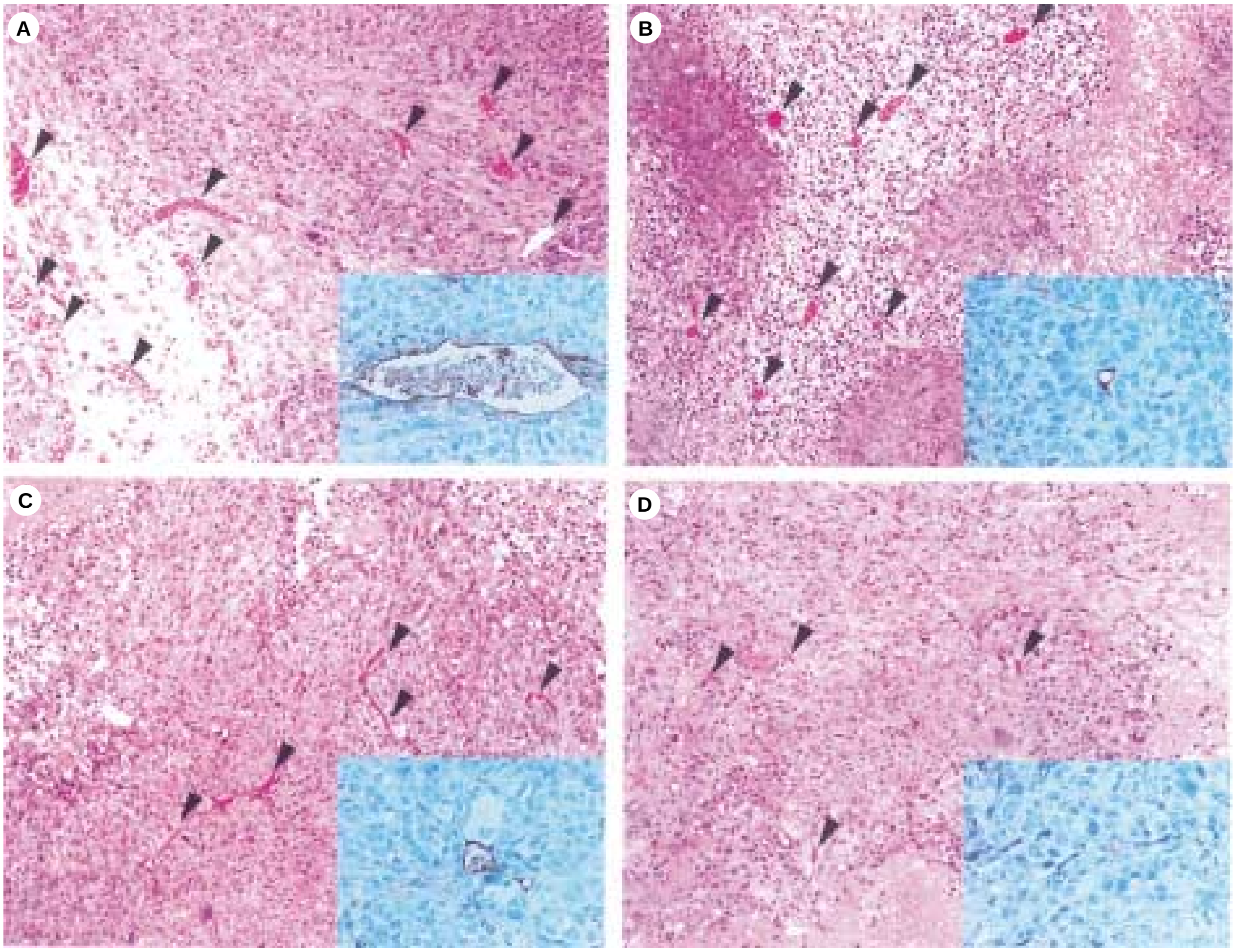

**Figure 4** Representative microphotographs of SW480 tumours (original magnification × 25) without treatment (**A**), with PTK787/ZK222584 treatment (**B**), irradiation (**C**) or combined treatment (**D**). Although a large number of distended microvessels (arrowheads) were detected, even at the border to the necrotic central parts of the control tumours (**A**), only a small number of thin microvessels were observed after combined treatment with PTK787/ZK222584 and IR (**D**). Tumours treated either with PTK787/ZK222584 (**B**) or IR (**C**) showed comparable microvessel density to control tumours (**A**). The insets display a representative vessel stained with an endothelial cell-specific anti-CD31 antibody (original magnification ×400)

effects of radiotherapy (Geng et al, 2001). Our results support the antitumoral therapeutic strategy of combining small, specific inhibitors of intracellular protein kinases with irradiation (Zaugg et al, 2001). In contrast to SU5416, PTK787/ZK222584 is active after oral administration and displays an increased specificity towards VEGF receptor tyrosine kinases compared to SU5416 (Fong et al, 1999; Wood et al, 2000).

Treatment with PTK787/ZK222584 and irradiation might cooperate on various levels with regard to tumour control. Irradiation targets both tumour and endothelial cells and as part of a stress response also enhances the expression of VEGF in the tumour tissue (Gorski et al, 1999). PTK787/ZK222584 downregulates the VEGF-dependent proliferative activity of the tumour microvasculature and concomitantly blocks the enhanced VEGF-mediated survival processes in response to irradiation (Gorski et al, 1999; Geng et al, 2001). Thus the molecular analysis of VEGF-dependent survival pathways in endothelial cells and the interaction with the irradiated tumour tissue will be of great importance to advance this rapidly developing concept of a cooperative tumour growth control effect by angiogenesis inhibitors in combination with irradiation.

### ACKNOWLEDGEMENTS

This work was supported in part by grants from the Ida de Pottère-Leupold (CH), Hartmann-Müller, Radium Foundation, Stiftung zur Krebsbekämpfung (OR), Swiss Cancer League (SB). The authors thank Angela Tenzer for statistical analysis.

### REFERENCES

Asano M, Yukita A, Matsumoto T, Kondo S and Suzuki H (1995) Inhibition of tumour growth and metastasis by an immunoneutralizing monoclonal antibody to human vascular endothelial growth factor/vascular permeability factor-121. *Cancer Res* **55**: 5296–5301

Borgestrom P, Hillan KJ, Sriramarao P and Ferrara N (1996) Complete inhibition of angiogenesis and growth of microtumors by anti-vascular endothelial growth factor neutralizing antibody; novel concepts of angiostatic therapy from intravital videomicroscopy. *Cancer Res* **56**: 4032–4039

Brown LF, Berse B, Jackman RW, Tognazzi K, Manseau EJ, Senger DR and Dvorak HF (1993a) Expression of vascular permeability factor (vascular endothelial growth factor) and its receptors in adenocarcinomas of the gastrointestinal tract. *Cancer Res* **53**: 4727–4735

Brown LF, Berse B, Jackman RW, Tognazzi K, Manseau EJ, Dvorak HF and Senger DR (1993b) Increased expression of vascular permeability factor (vascular

© 2001 Cancer Research Campaign 

endothelial growth factor) and its receptors in kidney and bladder carcinomas. *Am J Pathol* **143**: 1255–1262

Clauss M, Weich H, Breier G, Knies U, Rockl W, Waltenberger J and Risau W (1996) The vascular endothelial growth factor receptor Flt-1 mediates biological activities. Implications for a functional role of placenta growth factor in monocyte activation and chemotaxis. *J Biol Chem* **271**: 17629–17634

Drevs J, Hofmann I, Hugenschmidt H, Wittig C, Madjar H, Muller M, Wood J, Martiny-Baron G, Unger C and Marme D (2000) Effects of PTK787/ZK222584/ZK 222584, a specific inhibitor of vascular endothelial growth factor receptor tyrosine kinases, on primary tumour, metastasis, vessel density, and blood flow in a murine renal cell carcinoma model. *Cancer Res* **60**: 4819–4824

Folkman J (1995) Angiogenesis in cancer, vascular, rheumatoid and other disease. *Nat Med* **1**: 27–31

Fong TA, Shawver LK, Sun L, Tang C, App H, Powell TJ, Kim YH, Schreck R, Wang X, Risau W, Ullrich A, Hirth KP and McMahon G (1999) SU5416 is a potent and selective inhibitor of the vascular endothelial growth factor receptor (Flk-1/KDR) that inhibits tyrosine kinase catalysis, tumour vascularization, and growth of multiple tumour types. *Cancer Res* **59**: 99–106

Geng L, Donnely E, McMahon G, Lin PC, Sierra-Rivera E, Oshinka H and Hallahan DE (2001) Inhibition of vascular endothelial growth factor receptor signaling leads to reversal of tumour resistance to radiotherapy. *Cancer Res* **61**: 2413–2419

Goldman CK, Kendall RL, Cabrera G, Soroceanu L, Heike Y, Gillesie GY, Siegal GP, Mao X, Bett AJ, Huckle WR, Thomas KA and Curiel DT (1998) Paracrine expression of an native soluble vascular endothelial growth factor receptor inhibits tumour growth, metastasis, and mortality rate. *Proc Natl Acad Sci USA* **95**: 8795–8800

Gorski DH, Mauceri HJ, Salloum RM, Gately S, Hellman S, Beckett MA, Sukhatme VP, Soff GA, Kufe DW and Weichselbaum RR (1998) Potentiation of the antitumor effect of ionising radiation by brief concomitant exposures to angiostatin. *Cancer Res* **58**: 5686–5689

Gorski DH, Beckett MA, Jaskowiak NT, Calvin DP, Mauceri HJ, Salloum RM, Seetharam S, Koons A, Hari DM, Kufe DW and Weichselbaum RR (1999) Blockage of the vascular endothelial growth factor stress response increases the antitumor effects of ionising radiation. *Cancer Res* **59**: 3374–3378

Hanahan D and Folkman J (1996) Patterns and emerging mechanisms of the angiogenic switch during tumorigenesis. *Cell* **86**: 353–364

Joukov V, Kaipainen A, Jeltsch M, Pajusola K, Olofsson B, Kumar V, Eriksson U and Alitalo K (1997) Vascular endothelial growth factors VEGF-B and VEGF-C. *J Cell Physiol* **173**: 211–215

Kanai T, Konno H, Tanaka T, Baba M, Matsumoto K, Nakamura S, Yukita A, Asano M, Suzuki H and Baba S (1998) Anti-tumour and anti-metastatic effect of human-vascular-endothelial-growth-factor-neutralizing antibody on human colon and gastric carcinoma xenotransplantation orthotopically into nude mice. *Int J Cancer* **77**: 933–936

Kim KJ, Li BB, Winer J, Armanini M, Gillett N, Phillips HS and Ferrara N (1993) Inhibition of vascular endothelial growth-factor-induced angiogenesis suppresses tumour growth in vivo. *Nature* **362**: 841–844

Kozin SV, Boucher Y, Hicklin DJ, Bohlen P, Jain RK and Suit HD (2001) Vascular endothelial growth factor receptor-2-blocking antibody potentiates radiation-induced long-term control of human tumour xenografts. *Cancer Res* **61**: 39–44

Kroll J and Waltenberger J (1997) The vascular endothelial growth factor receptor KDR activates multiple signal transduction pathways in porcine aortic endothelial cells. *J Biol Chem* **272**: 32521–32527

Lund EL, Bastholm L and Kristjansen PEG (2000) Therapeutic synergy of TNP-40 and ionising radiation: effects on tumour growth, vessel morphology and angiogenesis in human glioblastoma multiforme xenografts. *Clin Cancer Res* **6**: 971–978

Mauceri HJ, Hanna NN, Beckett MA, Gorski DH, Staba MJ, Stellato KA, Bigelow K, Heimann R, Gately S, Dhanabal M, Soff GA, Sukhatme VP, Kufe DW and Weichselbaum RR (1998) Combined effects of angiostatin and ionising radiation in antitumour therapy. *Nature* **394**: 287–291

Olofsson B, Pajusola K, Kaipainen A, Joukov V, Saksela O, Orpana A, Pettersson RF, Alitalo K and Eriksson U (1996) Vascular endothelial growth factor B, a novel growth factor for endothelial cells. *Proc Natl Acad Sci USA* **93**: 2576–2581

Olofsson B, Korpelainen E, Pepper MS, Mandriota SJ, Aase K, Kumar V, Gunji Y, Jeltsch MM, Shibuya M, Alitalo K and Eriksson U (1998) Vascular endothelial growth factor B (VEGF-B) binds to VEGF receptor-1 and regulates plasminogen activator activity in endothelial cells. *Proc Natl Acad Sci USA* **95**: 11709–11714

Park JE, Chen HH, Winer J, Houck KA and Ferrara N (1994) Placenta growth factor. Potentiation of vascular endothelial growth factor bioactivity, in vitro and in vivo, and high affinity binding to Flt-1 but not to Flk-1/KDR. *J Biol Chem* **269**: 25646–25654

Shibuya M, Yamaguchi S, Yamane A, Ikeda T, Tojo A, Matsushime H and Sato M (1990) Nucleotide sequence and expression of a novel human receptor-type tyrosine kinase gene (flt) closely related to the fms family. *Oncogene* **5**: 519–524

Shibuya M (1995) Role of VEGF-flt receptor system in normal and tumour angiogenesis. *Adv Cancer Res* **67**: 281–316

Sun L-Q, Li Y-X, Guillou L and Coucke PA (1998) (E)-2´-Deoxy-2´-(fluoromethylene) Cytidine potentiates radioresponse of two human solid tumour xenografts. *Cancer Res* **58**: 5411–5417

Takahashi T and Shibuya M (1997) The 230 kDa mature form of KDR/Flk-1 (VEGF receptor-2) activates the PLC-gamma pathway and partially induces mitotic signals in NIH3T3 fibroblasts. *Oncogene* **14**: 2079–2089

Teicher BA, Holden SA, Ara G, Korbut T and Menon K (1996) Comparison of several antiangiogenic regimens alone and with cytotoxic therapies in the Lewis lung carcinoma. *Cancer Chemother Pharmacol* **38**: 169–177

Terman BI, Dougher VM, Carrion ME, Dimitrov D, Armellino DC, Gospodarowicz D and Bohlen P (1992) Identification of the KDR tyrosine kinase as a receptor for vascular endothelial cell growth factor. *Biochem Biophys Res Commun* **187**: 1579–1586

UKCCR (1998) United Kingdom Co-ordinating Committee on Cancer Research (UKCCCR) Guidelines for the Welfare of Animals in Experimental Neoplasia 2nd edn *Bri J Cancer* **77**: 1–10

Wood JM, Bold G, Buchdunger E, Cozens R, Ferrari S, Frei J, Hofmann F, Mestan J, Mett H, O'Reilly T, Persohn E, Rosel J, Schnell C, Stover D, Theuer A, Towbin H, Wenger F, Woods-Cook K, Menrad A, Siemeister G, Schirner M, Thierauch KH, Schneider MR, Drevs J, Martiny-Baron G and Totzke F (2000) PTK787/ZK222584/ZK 222584 a novel and potent inhibitor of vascular endothelial growth factor receptor tyrosine kinases, impairs vascular endothelial growth factor-induced responses and tumour growth after oral administration. *Cancer Res* **60**: 2178–2189

Yamada Y, Nezu J, Shimane M and Hirata Y (1997) Molecular cloning of a novel vascular endothelial growth factor, VEGF-D. *Genomics* **42**: 483–488

Zaugg K, Rocha S, Resch H, Hegyi I, Oehler C, Glanzmann C, Fabbro D, Bodis S and Pruschy M (2001) Differential, p53-dependent mechanism of radiosensitization in vitro and in vivo by the protein kinase C-specific inhibitor PKC412. *Cancer Res* **61**: 732–738

*© 2001 Cancer Research Campaign*
